# Genome Chaos, Information Creation, and Cancer Emergence: Searching for New Frameworks on the 50th Anniversary of the “War on Cancer”

**DOI:** 10.3390/genes13010101

**Published:** 2021-12-31

**Authors:** Julie Heng, Henry H. Heng

**Affiliations:** 1 Harvard College, 16 Divinity Ave, Cambridge, MA 02138, USA; jheng@college.harvard.edu; 2 Center for Molecular Medicine and Genetics, Wayne State University School of Medicine, Detroit, MI 48201, USA; 3 Department of Pathology, Wayne State University School of Medicine, Detroit, MI 48201, USA

**Keywords:** evolutionary mechanism of cancer, Genome Architecture Theory, information management, karyotype coding, National Cancer Act of 1971, two-phased evolution model

## Abstract

The year 2021 marks the 50th anniversary of the National Cancer Act, signed by President Nixon, which declared a national “war on cancer.” Powered by enormous financial support, this past half-century has witnessed remarkable progress in understanding the individual molecular mechanisms of cancer, primarily through the characterization of cancer genes and the phenotypes associated with their pathways. Despite millions of publications and the overwhelming volume data generated from the Cancer Genome Project, clinical benefits are still lacking. In fact, the massive, diverse data also unexpectedly challenge the current somatic gene mutation theory of cancer, as well as the initial rationales behind sequencing so many cancer samples. Therefore, what should we do next? Should we continue to sequence more samples and push for further molecular characterizations, or should we take a moment to pause and think about the biological meaning of the data we have, integrating new ideas in cancer biology? On this special anniversary, we implore that it is time for the latter. We review the Genome Architecture Theory, an alternative conceptual framework that departs from gene-based theories. Specifically, we discuss the relationship between genes, genomes, and information-based platforms for future cancer research. This discussion will reinforce some newly proposed concepts that are essential for advancing cancer research, including two-phased cancer evolution (which reconciles evolutionary contributions from karyotypes and genes), stress-induced genome chaos (which creates new system information essential for macroevolution), the evolutionary mechanism of cancer (which unifies diverse molecular mechanisms to create new karyotype coding during evolution), and cellular adaptation and cancer emergence (which explains why cancer exists in the first place). We hope that these ideas will usher in new genomic and evolutionary conceptual frameworks and strategies for the next 50 years of cancer research.

## 1. Introduction

The year 2021 is the National Cancer Act’s 50th birthday [1]. The Act had a profound impact on scientific progress and status, shifting the landscape of cancer research. According to leading cancer gene researcher Robert Weinberg, “The molecular cancer story really began early in the decade—1971 to be precise—when an enormous pot of money suddenly became available for cancer research. President Nixon’s War on Cancer, as it came to be called, was fueled by the conviction that cancer was ultimately a disease of infectious tumor viruses.” [2].

However, as Weinberg describes, it soon turned out that the scientific conviction to start the war was wrong.

“Looking back, it’s clear that the scramble to find human retroviruses represented the major irony of the War on Cancer: it had been launched for the wrong reason, since cancer-causing human retroviruses were never found (with the exception of rare leukemias in the Caribbean and southern Japan).”

[2]

Nevertheless, as with many other complex adaptive systems, the war on cancer found its own life. Supported by renewed funding, new convictions arose and became new targeted battles with new rationalizations: from oncogenes to tumor suppressors, differentiation genes, DNA repair genes, cell cycle genes, cell death genes, metabolic genes, stress response genes, genome instability genes, and immune genes; from a handful of cancer genes to hundreds classified by “cancer hallmarks” to thousands of genes and beyond; from DNA to RNA to proteins, lipids, and sugars; and from cellular organelles to gap junctions to tissue structure. Although each battle promised to end cancer once and for all, the war goes on, seemingly endlessly [3,4]. 

These battles have faced highly diverse targets, although all share the same assumption: cancer is a disease of uncontrollable growth, with a causation that can be identified by studying gene mutation and epigenetic regulation. Then, when all these individual battles failed to deliver, a bold idea emerged as the final battle: can we sequence every single gene mutation in cancer? Therefore, the Cancer Genome Atlas (TCGA) was born. It promised to leave no stone unturned and finally understand and cure all cancers [3,5].

Meanwhile, after conducting “watching cancer evolution in action” experiments [6], we proposed that karyotype-mediated macroevolution, not gene mutation, is the common driving force for most cancers. Our genome-based theory (which would formally develop into the Genome Architecture Theory) predicted that large-scale sequencing would reveal high levels of data heterogeneity—in other words, a larger sample size could not solve our problems [5,7]. Unfortunately, despite efforts made by pioneers such as Richard Goldschmidt and Barbara McClintock, few researchers have appreciated the ultimate importance of genome-level changes [3,8]. According to the dogma of molecular genetics, the chromosome is just a carrier of genes. Most genetic errors are rare events due to multiple failures in error correction mechanisms such as DNA repair, cell cycle checkpoints, ER stress responses, information feedback, and apoptosis [8]. Such elegant and precise regulatory mechanisms resulted from billions of years of natural selection accumulating small changes over time; thus, punctuated, large-scale genomic aberrations must be harmful and cannot fit into the evolutionary process. Additionally, molecular genetics claims that drastically altered karyotypes, which are often observed among dying cells, cannot become clonal populations. Moreover, compared with genes, chromosome aberrations are harder to quantitatively measure for mathematical models, and efforts to identify common chromosomal aberrations for a majority of cancers have, thus far, failed [9]. 

Fast forward, the avalanche of sequencing data we have is at odds with the initial goal of TCGA, to identify a few shared cancer gene mutations. TCGA has exposed a highly heterogeneous genomic landscape that challenges the somatic gene mutation theory of cancer [3]: interestingly, (1) chromosomal alterations are overwhelming in most cancer types, and the profiles of chromosomal changes have much better clinical prediction power than those of gene mutations [10,11,12,13,14]; (2) for many patients, driver gene mutations are hard to identify, although benign tissue could have even more gene mutations than its malignant counterparts [15,16]; and (3) every gene is a relevant “cancer gene”, undermining TCGA’s original mission [17]. The disappointment of gene-based prediction is not limited to TCGA, however. GWASs have revealed many involved loci and few dominant positions [18,19]; experiments have illustrated that the lost function of specific genes can be recovered by aneuploidy [20]; high levels of genomic mosaicism are detected in somatic cells [21,22,23]; and somatic genomic and/or non-genomic dynamics are common during development, aging, and stress response processes. Altogether, the gap between gene profile and phenotype becomes wider, and the power of gene-based prediction is drastically reducing [8]. 

Obviously, genetics and genomics have now entered uncharted waters. Genes are still considered not only the basis of current genetic and evolutionary theories, but also fundamentally important for medical genetics, the driving force for diseases as well as organismal evolution. If the power of genes is less certain than we have imagined, how will the most dominant theories in biology and their biotechnological and medical implications be impacted? Additionally, equally importantly, how should we practice biology?

Recently, Nobel Laureate Paul Nurse suggested that biology is generating data without ideas to match [24]. In his opinion piece, Nurse quoted Sydney Brenner’s earlier warning for biology that “We are drowning in a sea of data and starving for knowledge.” Clearly, the gap between data and explanation has increased rapidly due to various large-scale -omics platforms, including single-cell technologies, and this gap will only be exacerbated in the future. Nurse recommends a shift in the culture of scientific research by emphasizing ideas and theories in addition to data generation, because current biology lacks the theories to explain or even generate data, as well as worthwhile ideas to inspire new generations. Although the piece is a timely reminder for molecular biologists in the era of Big Data that data and ideas should go hand in hand, Nurse did not address the issue of why technically orientated ideas differ from key theories of biology. Moreover, we must ask ourselves why and when more data means less understanding. What must we do when current theories and data conflict [5,8]?

Supposedly, more data helps us understand more. However, this statement is only true if the data are relevant to the question being studied and the data are explainable within our scientific theory. For example, we usually do not try to classify different species based on the behavior of their atoms. According to Thomas Kuhn, during a routine phase of science, when most data make sense, science progresses. However, when data obviously conflict with the theories that guide the efforts of data generation and collection, alternative theories may be necessary. Quickly reviewing some major cancer genome sequencing data published from the top journals, much of the field’s data can be explained although by neither the somatic gene mutation theory nor neo-Darwinian evolution. Interestingly, researchers often avoid pointing out such inconsistencies or seem altogether unkeen in discussing ideas and theories. 

Interestingly, alternative theories that better explain our “surprising” cancer gene mutation data exist, though there are no common frameworks to unify them (Table 1).

Nevertheless, many of these different ideas are suppressed by the mainstream research community. This suggests that we do not lack ideas/observations, but lack new frameworks to hone and mature these alternative ideas. In other words, we might have reached a moment of scientific revolution. The current lack of new ideas and the difficulties in solving key anomalies represent key features of a crisis, a necessary stage of a paradigm shift: “One of the key preconditions and signals of a paradigm shift is the transition from a routine progression stage to a crisis stage of a given scientific field. In a real crisis stage, the dominating paradigm is losing its capability to explain fundamental facts (most of which are newly discovered), despite that there are many superficial technical achievements being made and a large amount of data being collected. In other words, the more data that are collected, the more confusion there is and the less we can comprehend it, as these new discoveries contradict the expectations of the current paradigm. Such increased anomalies are highly unfit between the existing theory and reality.” [8].

After 50 years of war, does current cancer research need a new paradigm? If yes, what should it look like? How much do cancer theories and research strategies need to be altered? Our key predictions about the outcome of the Cancer Genome Project have proven to be correct [5,7], and we believe it is time to renew the call to invoke a new genomic and evolutionary framework [8]: the Genome Architecture Theory (GAT). The GAT (previously referred to as the Genome Theory or Genome System Theory) departs from the somatic gene mutation theory of cancer [5,7,8,84,86]. In this perspective, genetic and genomic concepts are briefly compared, and some newly emergent principles of the GAT are clarified in the context of inheritance, information, and evolution patterns. Finally, some lessons are offered from the war on cancer. 

## 2. Newly Emergent Genomic and Evolutionary Concepts

### 2.1. Genes vs. Karyotypes (Chromosome Sets): Redefining Inheritance

The transition from studying karyotypes to genes once represented a technological advancement in cancer research [54]. Based on reductionist practice, the higher the resolution, the better the experimental approach. As soon as fusion genes could be isolated from translated chromosomal regions, karyotype analyses became less important. Compared with gene studies, karyotype studies were often dismissed as “not mechanistic enough” and relegated to the less fundable “descriptive studies” category. 

However, following 50 years of gene-based cancer research, especially after the Cancer Genome Project, increased gene data have unexpectedly illustrated the complexity and uncertainty of cancer, which forcefully challenges some of the key assumptions of the somatic gene mutation theory. For example, the gene was assumed to be an independent information unit, where key genes are responsible for key traits. It followed that cancer genes would be common drivers for cancer evolution. Why, then, were sequencing results data and theoretical predictions so far off? Among many explanations (see [3,5,8]), two key conceptual misunderstandings of genetics and evolution are worth re-emphasizing: 

(1). The gene’s predictive power has been artificially overestimated by researchers through their selective experimental systems and data interpretations. 

As we have previously discussed [7,8], clear-cut relationships between genes and phenotypes are often only observable in exceptional cases. Artificial experimental systems and selective datasets have been key to establishing the power of single genetic elements or genes, ever since Mendel’s foundational experiments. Not only it is difficult to repeat Mendel’s experiments using different species, but he also only reported seven traits among the many he had examined. When studying each trait, his extreme selection of individuals played a major role, choosing uncommon stem lengths for example:

“Third, Mendel had a strict selection criterion for each sample. He had purposely avoided collecting “average data” by using exceptional samples in his experiments. For example, to compare the difference in the stem length (one of his 7 traits), a long axis of 6–7 ft was always crossed with a short one of 0.75–1.5 ft. By pushing extreme cases rather than using average long and short populations, the certainty of data becomes much more impressive. Paradoxically, however, the pattern he discovered based on selection will not represent the majority of the data he ignored.

Fourth, Mendel had tried his best to reduce environmental variations that could influence the data, such as growth conditions, the timing of experiments, and the effect of all foreign pollen, which invariably created ideal systems with minimal environmental influences.

Together, Mendel had created a perfect yet highly exceptional system. Perfect for a manipulated linear model with reduced variants, exceptional for the reality of genetics where most genetic traits do not contribute by a single gene and heterogeneity dominates within a population.” [8].

Knowing how Mendel studied the genetic basis of the length of pea stems, we can understand why it has been so difficult for current researchers to study the genetic basis of traits such as human height despite better technologies and sample sizes. Mendel likely would have faced similar difficulties had he used the entire set of stem lengths in the pea plant population. The genetic basis of human height could be much simpler if we only examine extreme “giant” and “dwarf” phenotypes, because an excess or deficiency of growth hormone would be the obvious cause. However, such a simple answer will not explain the majority of differences in individuals’ heights. Recently, more than 100,000 genomic variants affecting human height were identified, most of which were previously considered statistical noise and ignored; these variants are less useful for pattern identification because they are distributed across the entire genome [18].

Even so-called Mendelian diseases, which are purportedly caused by a single gene, involve many other genes, such as multiple levels of modifiers, and genes related to system function, including homeostasis. The same “causative” mutation can cause different phenotypes across individuals.

The real challenges for future genomics are the facts that the power of any gene is limited and that genes do not function individually but within complex networks. Furthermore, gene-coded information is fuzzy, and environments can “select” outcomes among many coded potentials [8]. By introducing linear, causative experimental systems, researchers often can “prove” the predictive power of a gene under study, but these linear relationships fall apart in a natural setting, where uncertainty is much higher. It is thus no surprise that so many genes—nearly all genes—can be linked to cancer formation and growth [17]. Now, the more serious question becomes: if (almost) every gene affects (almost) everything, how does genetics actually work [8]?;

(2). The mechanisms of inheritance in macro- and microevolution have traditionally been misunderstood, resulting in much confusion in cancer research

Since the gene was identified as a basic unit of inheritance, the research landscape of modern biology has been dominated by studying how genes program cellular phenotypes through various bioprocesses. Successful efforts include characterization of the gene itself (structure, replication, repair) and its relationship with RNA, proteins, and other biological components/pathways; understanding gene regulation and function in developmental as well as normal physiological processes; gene frequency dynamics within a given population; and the cloning of genes responsible for inherited diseases, including some familial cancers (however, for most sporadic cancers, gene theory has lost much of its explanatory power). 

Confusion about the power of genes arises from misunderstandings of gene-defined inheritance and cancer. First, Mendelian genetics is objectively constrained to study genetic processes occurring within a given species. Second, since the beginning of molecular cancer research, cancer has been defined as a gene mutation problem, where the oncogene can promote cellular proliferation. Both these assumptions fail to realize that cancer is a new system emergent from multiple levels of system constraints. Theodor Boveri linked cancer to chromosomal aberrations, the only genetic mechanism he knew, in 1902 [87]; since then, molecular geneticists have concluded that the gene is a more advanced concept than the chromosome, because the chromosome is just a vehicle carrying genes. As a result of the dominance of genes, few molecular geneticists are interested in examining the impact of an altered karyotype on their favored gene pathways. However, crucially, the karyotype plays a key role in organizing gene interactions and preserving system information (for more details, refer to later sections). In other words, karyotype change generates a new system. Furthermore, our decades-long research has linked karyotype change with “system inheritance”, which mainly involves macroevolution, and gene mutation with “parts inheritance”, which involves microevolution, including normal developmental processes [7,8,88].

The importance of the karyotype was also ignored by most biologists who use model systems to study gene function. In these systems, the karyotype is often maintained; therefore, the function of a gene can be observed across various experimental settings. In cancer evolution, in contrast, the function of a gene is often indecipherable amid karyotype changes that impact many genes and the networks among them [3,8].

In essence [3,8], current confusions in cancer research are rooted in misunderstandings of inheritance and evolution. The purpose of this piece is to call for the re-examination of basic biological theories to rethink the cancer issue. For example, if the gene and karyotype represent two different types of inheritance, and cancer is an issue of system emergence with a new genome, monitoring the karyotype is an obvious necessity, especially when exorbitant gene-based research has failed to deliver its promises.

### 2.2. Molecular vs. Evolutionary Mechanisms: The Parts/Pathways, the System, and the Selective Processes

Research strategies are heavily influenced by theories. Since the identification of the first oncogene, the characterization of parts (e.g., identifying and analyzing cancer genes and proteins) has been the main focus of cancer research. When many components of the accumulated data conflicted with each other, various -omics approaches and systems biology were introduced. However, although overwhelming complexity in cancer has been confirmed, there are few specific suggestions of how to proceed. Researchers favor different approaches, including sequencing more samples of genomic and epigenetic landscapes for different cancer types, targeting various pathways, analyzing micro-RNA/non-coding RNA, studying metabolic contribution and influence of microbiota, and examining options of immunotherapy.

Many factors, genomic and environmental alike (from gene to lifestyle), are involved in cancer [3,4]. Under different experimental systems and conditions, any and all factors can promote, slow, or stochastically alter the cancer progression landscape; a given gene can switch from a tumor suppressor to an oncogene function or vice versa; the outliers can multiply into a dominant population or a key population can be eliminated; and a defined, specific pathway can soon become unpredictable when further variants are introduced. These data, saturated with complexity and uncertainty, force researchers to ask some important questions: What is the clinical value of studying one specific gene mutation within a highly adaptive system?; When data components conflict with each other, how do we choose which component to prioritize? 

To address these questions, one first needs to understand the relationship between different molecular mechanisms and the common mechanism of cancer [89,90]. Cancer is an evolutionary process; therefore, it must involve the generation of variations in the cellular population (which involves information creation at genome and gene-level); these variations display different degrees of reproduction, survival, and/or evolvability. These variations must be heritable. Furthermore, these altered cellular systems need to respond to evolutionary stress and must ultimately break environmental constraints. Then, any individual molecular mechanism that can contribute to the cancer evolutionary process can be linked to cancer.

However, although there are many specific and highly diverse gene-mediated molecular mechanisms, these can be unified by the common mechanism of genome-mediated cancer evolution under the evolutionary mechanism of cancer.

During comparative studies of tumorigenicity, in which five different well-characterized model systems were used, the degree of karyotypic heterogeneity (population diversity) was directly linked to tumorigenicity. The tumorigenicity of each model has been linked with different and specific molecular pathways and there is no common molecular mechanism shared among them; therefore, we realized that the common link of tumorigenicity between these diverse models is elevated genome diversity. Based on the concept that genome-level heterogeneity is a key to cancer evolution and that stress can induce increased system dynamics, reflected as increased NCCA frequencies, we proposed that the evolutionary mechanism of cancer is equal to the sum of all individual molecular mechanisms:

**Evolutionary Mechanism = ∑ Individual Molecular Mechanisms**

Although there are many individual molecular mechanisms, there are four key components for understanding how the evolutionary mechanism works: (1) stress-induced system dynamics (e.g., genomic, epigenomic, and increased stochastic changes); (2) population diversity (genome heterogeneity, which can be triggered by diverse gene mutations or molecular pathways when the stress is sufficiently high); (3) selection based on the genome package (macrocellular evolution); and (4) new genome systems capable of breaking down of higher levels of constraints and becoming the dominant population [8].

With the above realization, we now understand the limitation of focusing on the partial characterization of individual gene mutations, because there are so many, and most are trivial compared with karyotype alterations. Instead, we need to focus on evolutionary selection based on new emergent systems (cellular populations with new karyotypes). In addition, the unity of cellular macroevolution occurs at the genome level, rather than at the gene level. Gene mutations are mainly involved in the microevolutionary phase. 

Changes in research strategy also reflect general trends in biological research. At the initial stage of molecular research, researchers only knew to study isolated parts. Currently, researchers are struggling to integrate these well-studied parts, which requires holistic concepts and tools. In the future, researchers need to focus on profiling the evolutionary selection process, perhaps through watching-evolution-in-action experiments [6]. For example, over the course of a cancer treatment, it could be tolerable not knowing specific potential pathways, which can be quickly replaced by another pathway when the system undergoes a highly dynamic adaptation. Instead, we ought to focus on better predicting a system’s behavior and modifying its general trends. 

### 2.3. Genome Chaos: The Essential Process of System Information Self-Creation

The main reason why cancer often wins battles in the war on cancer is its incredible evolvability. Ironically, this evolvability is greatly enhanced by our medical treatment strategies. In other words, in the name of killing monsters, we may be propagating them more, because treatment options designed for the maximal killing of cancer cells can result in the formation of more aggressive cancers via genome chaos.

Genome chaos, rapid and massive genome re-organization under crisis, was initially systematically described during watching-evolution-in-action experiments [6,70,91]. It was soon realized that genome chaos is essential for key phase transitions in cancer evolution, including immortalization, transformation, metastasis, and drug resistance [3,8]. Chaotic karyotypes had occasionally been observed by cytogenetics, but they were largely ignored without the foundation of specific mechanisms and experimental systems to reproducibly generate them. Even after our initial report, suspicions remained high, based on the general view that: (1) there is no chance for these drastically altered structures to survive, and they have no evolutionary significance because the evolutionary selection is based on the accumulation of the small changes, and (2) high genetic integrity will not tolerate such structures. Thus, any chaotic genomes belong to genetic noise, which will certainly be eliminated by evolutionary selection.

In contrast, unexpectedly, chaotic genomes have commonly been detected from cancers in the Cancer Genome Project [92,93,94]. Although many different new terms were employed to describe the phenomena, previous cytogenetic discoveries of genome chaos were confirmed. As described in our previous publication:

“Recently, the cancer genome sequencing project has generated large amounts of data, which inevitably confirmed the importance of studying genome chaos [95,96,97]. Various chaotic genomes were detected within nearly all types of cancers. In some cancer types such as prostate cancer, chaotic genomes were detected in a majority of cases. Interestingly, these fragmented and stitched chromosomes were given many different names by different investigators including “chromothripsis”, “chromoplexy”, “chromoanagenesis”, “chromoanasynthesis”, “chromosome catastrophes”, and “structural mutations” [98,99,100,101,102,103,104,105,106,107,108]. Based on their descriptions chromothripsis refers to the chaotic genome mainly involving local re-organization (within a single chromosome), while chromoplexy refers to a more whole genome re-organization involving many individual chromosomes. We prefer using “genome chaos”, “karyotype chaos”, or “chromosomal chaos” to refer these structures due to their broad coverage (from local to global re-organization, from structural to numerical changes) and the simplicity of terminology [3,109,110,111].

Limited mechanistic studies mainly focused on specific gene mutations, pathways, and specific cellular mechanisms, such as how the p53 mutation and micro-nucleus formation contribute to genome chaos. For example, it was elegantly illustrated that chromothripsis may directly originate from DNA damage in micronuclei, linking the restriction of chromothriptic rearrangements to a single chromosome. Alternatively, chromothripsis was linked to telomere crisis [112,113]. From a genome evolutionary perspective, however, there should be large numbers of specific molecular mechanisms that can contribute to genome chaos, and only a small portion of them belong to chromothripsis [3,6,114]. Additional studies have now linked many individual factors, such as hyperploidy and radiation, to chromothripsis [115,116], and the list of the contributing factors should be increasing…” [117].

Following the tradition of molecular research, increased attention has focused on the molecular mechanisms for different subtypes of genome chaos. In a recent comprehensive review article, James Shapiro summarized different molecular mechanisms that are responsible for different chaotic genome subtypes [92]. Although it is interesting to study the individual mechanisms for chromothripsis, chromoplexy, micronuclei clusters, and many other structural and numerical chaotic genomes, such mechanisms unhelpfully involve nearly unlimited pathways and trigger factors [4,114]. There is now increased attention on polyploid giant cancer cells (PGCCs), a numerical chaotic genome subtype, in cancer’s aggressiveness and drug resistance [71,72,73,74,75,76,91,109]. Due to the direct relationship of the different phases within the cell cycle (a defect in the S phase can impact the G2, M, and G1 phases), various types of chromosomal abnormalities are linked (replication defects can lead to the error of chromosomal condensation and segregation, or vice versa) [55]. Furthermore, under the stress response, many subtypes of chaotic genomes are intimately linked by a “cause and consequence cycle”, and can constantly alter subtypes [54,70,110,118]. More challengingly, it would be even harder to study or predict the dynamics of multiple variants during active evolutionary selection. A more practical approach is to focus on the evolutionary mechanism of cancer: regardless of the individual trigger factors and mechanisms of this phenomenon, the common evolutionary consequence is the same—to produce the new genomes that are essential for phase transition during the evolution [8,53,118,119].

Two useful concepts can help one truly appreciate the importance of genome chaos: First, chaos theory is a new frontier of science that differs from the layman’s definition of disorder. Although genome chaos represents a highly heterogeneous process, the outcome of this process is predictable. For example, myriad triggers (uncertain factors) can generate high stress, and the cellular program of genome re-organization will be initiated (a certain consequence). However, the cellular system cannot guarantee the production of only winning karyotypes—it always produces massively different karyotypes (with uncertainty). Then, evolution picks the winners. Given enough chance, there will be winners (certainty), but who the winner will be is unknown (uncertain). That is why it is less useful to focus on any specific pathways. As we have stated:

“Chaos as a system behavior does not simply mean random disorder. Why use the term “chaos” to describe genome-based rapid cancer evolution? Genome behavior during crises shares common features with complex systems described by chaos theory. Chaos theory, a non-linear dynamics concept, states that “within the apparent randomness of chaotic complex systems, there are underlying patterns, interconnectedness, constant feedback loops, repetition, self-similarity, fractals, and self-organization” (https://en.wikipedia.org/wiki/Chaos_theory) (accessed on 12 August 2021). It is important to note that, within the collective frameworks of chaos theory, apparently random states of disorder and irregularities of dynamic systems are often governed by deterministic laws. In layman’s terms, unfortunately, the word “chaos” suggests rampant disorder and randomness, coupled with a negative connotation”

“…Genome chaos represents a process that can be described by a combination of uncertainty and certainty, disorder and order, stochasticity and determinism—an excellent example of random means to a nonrandom end function. Therefore, analyzing system behavior (how macroevolution is achieved by the success of creating new systems), rather than characterizing all involved genome variation mechanisms, will make the cellular macroevolution phase transition much easier to understand and predict.” [119];

Second: genome chaos research has led to the important concept of System Information Self-Creation Under Crisis, which will have a profound impact on cancer research and evolutionary biology [120]. Genome reorganization and role driving phase transitions in cancer led us to search for “system inheritance”-coded “system information”, distinctive to “parts inheritance”-coded “parts information.” Such efforts have established the concept of karyotype coding: 

“‘Karyotype coding’ or ‘chromosomal (set) coding’ functions as an organizer of gene interactions within the entire genome. Its biological effect is not just on individual genes but on the entire genomic network. As opposed to gene coding or vague ideas that chromosomes carry additional information, karyotype coding is defined by specific features: (1) the physical organization of the chromosome codes system information; (2) genomic topology provides context for individual genes; and (3) since different species display unique karyotypes or core genomes, karyotype coding is often species-specific. The key is that the order of gene and non-coding sequences along a chromosome represents a new ‘system inheritance,’ much like how the order of base pairs codes for ‘parts inheritance’ in mainstream ‘gene coding.’” [86].

There are many obstacles for most gene-based researchers to accept karyotype coding. The most significant one is the constraint of the somatic gene mutation theory, which is not compatible with genome-based theories. Another key factor is a lack of understanding of the multiple levels and types of bio-information. According to reductionist tradition, genetic information solely means gene-coded information, and the system information that organizes gene-level information is irrelevant. The Genome Architecture Theory classifies information management into information creation, preservation, modification, and usage [53,119,120]. Different types of coding have been linked to different types of inheritance, and karyotype coding is crucial for preserving combinational self-organization events (including chemical and physical events). Karyotype coding serves as a platform on which other organic codes can operate and accumulate, leading to increased biocomplexity and diversity [120,121,122,123].

Perhaps most importantly, new system information creation in biology is a process of self-creation. Under high-stress conditions likely to eliminate a system, the system’s cellular machinery will automatically switch into a mode that destroys the current genome and simultaneously forms new genomes using their own genomic materials. Changing the karyotype coding represents the only opportunity to pass on the information of life, at a level above individual cellular species. This capability of creating new from self-death is witnessed in rapid and massive drug-treatment-induced genome chaos, which produces drug resistance. As newly formed systems with new karyotype coding, these resistant individuals are no longer their original “selves.” Such a mechanism not only explains the basis for treatment-induced drug resistance [70,71,76,91], but also explains organismal macroevolution, especially when large numbers of new species emerge from massive extinction [8,124], (Heng et al., submitted). Of course, organismal evolution is more complex than cancer evolution. Somatic evolution selects stable genomes among many created unstable ones, and stable genomes are key to preserving system information. Interestingly, karyotype preservation is achieved in animals and plants through sex and the separation of the germline and somatic cells during development [8,63,125,126].

### 2.4. Two-Phased Evolutionary Model

**Figure 1:**
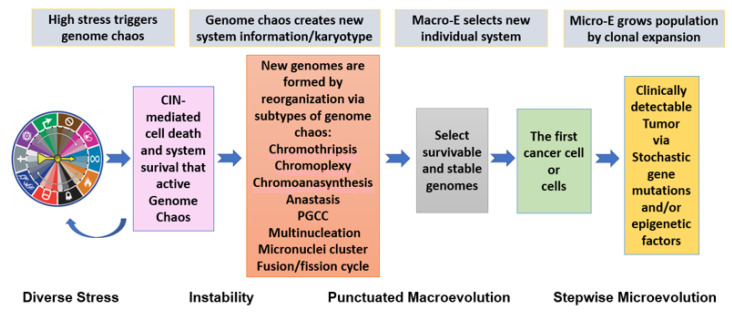
**New model of two-phased cancer evolution**. Diverse stresses are represented by the hallmarks of cancer (modified from [90,127]). When stress is high enough to kill cells, it can trigger genome chaos. Genome chaos can manifest as many subtypes, both structural and numerical, of which only seven examples are listed here. Regardless of the individual subtypes, new system information is created. Evolution selects the first cancer cell or the first wave of cancer cells, which are then subject to microevolution to grow the cancer cell population. This process can be linked to large numbers of different gene mutations or pathways. For more information, please refer to [3,4,8,128].

A direct benefit of synthesizing all the above information (from redefining inheritance to illustrating genome chaos as a process for creating new system information) is to establish the model of two-phased cancer evolution. This model comprises a punctuated phase and a gradual stepwise phase. Within the punctuated phase, karyotype changes dominate, and genome-based macro-selection is the key driving force. Within the gradual phase, gene mutations and epigenetic alterations dominate, with microevolution as the key feature. This model can reconcile the contribution of genes as well as karyotypes. It is also useful for both cancer diagnosis and treatment [3,6,8,53,76,120]. As illustrated in Figure 1, the model of two-phased cancer evolution can acceptably integrate and unify stressors, various subtypes of genome chaos, system information creation by genome re-shuffling, the macroevolutionary selection of survivable new genomes, the clonal expansion of the “first cell”, and the involvement of immense gene mutations/epigenetic abnormalities that contribute to microevolution.

## 3. Future Perspective

Clearly, the National Cancer Act has significantly altered the landscape of cancer research. It set in motion a chain of events, including government funding, infrastructure (the National Cancer Program), the promotion of bioindustry, and the National Cancer Institute (NCI)’s influence on the direction of national research, which has greatly promoted basic research. In addition to millions of manuscripts published and thousands of genes identified, a large body of molecular knowledge and experimental platforms/methodologies have been accumulated. Some impressive successes have also been achieved, such as imatinib, a drug for CML; an HPV vaccine for cervical cancer prevention; and the current development of immunotherapy. Interestingly, the data generated from the National Cancer Act have also unexpectedly sped up the effort to search for new frameworks beyond the gene, because the well-funded cancer field has accumulated so much molecular data that forcefully challenge many current biological theories [3,8].

Here are some further lessons learned in the past 50 years of the war on cancer. 

First, the war metaphor has had unexpected consequences on researchers, physicians, and patients. The war mentality promotes a host of combative strategies against cancer. Treatments prioritize killing the enemy at all costs, using the most powerful weapons possible [76]. Combined with the rhetoric and ideas of searching for magic bullets and specific targets, the entire field has spent too much effort on molecular details in order to increase treatment certainty. Cancer is a complex adaptive system with evolution as its key mechanism; therefore, the current approach of understanding all possible molecular agents and systematically fighting them is both impossible and less useful.

Second, conducting basic science differs from finishing an engineering project, especially when the science is undergoing a framework change. For a defined engineering project, if there is a solid theoretical framework and implementable technologies, sufficient funding and labor can deliver the products, whether this is a nuclear bomb or moon landing. To cure an unknown, which requires both new theories and technical platforms, fulfilling a goal within a fixed amount of time will likely fail. The Human Genome Project (HGP) is a good example. Yes, we can sequence a human genome on time with improved technologies (an impressive engineering project), but we should not promise that we can decode the mystery of life and eliminate all diseases in our lifetime with said sequencing project (basic science). 

During the past 50 years, both governments and research institutions have promised to cure cancer many times, to no avail [3]. From a promising consensus mentioned by the National Cancer Act of 1971: “There seems to be a consensus among cancer researchers that they are within striking distance of achieving the basic understanding of cancer cells which has eluded the most brilliant medical minds in the world” [1]; to President Bill Clinton’s statement in 2000, following the celebration of the success of the HGP: “It is now conceivable that our children and our children’s children will know the term cancer only as a constellation of stars…Genome science… will revolutionize the diagnosis, prevention and treatment of most, if not all, human diseases” [129]; to the NCI’s declaration that cancer will be cured by 2015; to President Obama’s promise in his 2016 State of the Union address: “For the loved ones we’ve all lost, for the family we can still save, let us make America the country that cures cancer once and for all” [130]; and to the proposed Moonshot to cure cancer in 2023 by a well-known cancer center [3,8], we witness one empty promise after another. Nuance is necessary here: optimism is good, but overstatements harm both scientists and patients. 

Third, biology in general, and cancer research in particular, needs solid theories. We already have overwhelming data. Theoretical analyses and the re-examination of current concepts are urgently needed before we further push any data generation. For example, although single-cell data are interesting and potentially valuable, the system behavior of a cellular population is an emergent one, with contributions from both the average and outliers. Thus, we cannot study any disease by only using the averages of a population. Without the correct theoretical framework, without noticing the impact of outliers, we fail to see the big picture. Instead, we will only produce more data without biological insight, attributing the effects of outliers to the average. 

As for the re-examination of basic theories in biology, both genomic and evolutionary theories should be prioritized. Specifically, how genes and karyotypes contribute to parts and system inheritance in cancer research, and the common pattern of cancer evolution, need more attention. It is important to separate gene mutations and chromosomal alterations in cancer research and diagnosis, because these alterations can be used as indices to monitor the different phases of cancer evolution. In addition, the multiple levels of genomic and non-genomic heterogeneity can be investigated using the concept of fuzzy inheritance [8]. Furthermore, heterogeneity, especially at the chromosomal level, can be used to monitor and/or predict general trends of cancer evolution.

The past 50 years in particular, with the Cancer Genome Project, have already produced sufficient data. What we need is to perform a systematic comparison to validate the model of two-phased cancer evolution and apply it to cancer diagnosis and treatment. For example, employing a moderate force of constraint in the microevolutionary phase could slow down cancer microevolution evolution without triggering genome chaos that would rapidly produce drug-resistant genomes. If successful, this approach can be integrated into adaptive therapy strategies [131].

Other theories, in the realms of information theory and complex system theory which originate in non-biological systems, need to be further developed to fit the biological context of cancer [120]. Currently, most cancer researchers still focus on gene-coded parts information and favor linear models to study cancer mechanisms. When applying information theory to study cancer, the first question should be: What types of information we should collect?; How much is minimal?; Additionally, when different types of information conflict, how do we prioritize them? When applying complex adaptive system theory in cancer research, researchers should know that the certainty illustrated in a linear model has very limited clinical value because the causative relationship they demonstrate is likely an illusion under simplistic experimental conditions. 

One related issue is the misinformation perpetuated by the misguided use of preclinical assays. For example, even though the Nomenclature Committee on Cell Death has been warning the scientific community about the misuse of words and concepts that slow down progress in cancer research [132], most researchers simply ignore these caveats. Now it has become clear that the short-term benefit of induced cell death can paradoxically promote cancer, because dying cancer cells can emerge from the brink of death through genome chaos, including Anstasis (a cell recovery phenomenon that rescues cells from the brink of death) and PGCCs [8,71,76,77,91,117]. This can be explained as an example of why linear experimental models often cannot predict clinical realities when multiple types of evolution are involved.

In order to promote questioning of the current paradigm, as well as the creation of more accurate paradigms, our scientific culture needs to change. Institutionally, the NIH should promote theoretical study. Various organizations should actively promote much-needed debates to provide a competitive landscape for different ideas and approaches; for example, reviewers and grant agencies should ask authors to spell out the meaning of their presented data to see whether the data conflict with or support their initial rationale. Similarly, the NCI needs to discourage data generation without good ideas, because it is unwise to characterize all genes that can be linked to cancer—there are simply too many.

Moreover, we must increase education for scientists, physicians, and patients. In the future, it is essential to educate patients that cancer represents an evolutionary trade-off of cellular adaptation. To achieve various cellular functions (e.g., tissue repair, protection against bacterial infections), cells need to be able to change through cellular adaptation. However, such changes (genomic or non-genomic), although important for cellular function, can lead to various diseases, including cancer. Many genomic and environmental factors can contribute to cancer, and this becomes the main challenge for studying cancer genes for clinical use, because all these factors contribute to cancer but have very limited prediction power in the clinic. This evolutionary process is very complex and features high uncertainty; thus, the best method for individuals to prevent cancer is general lifestyle improvement. During treatment, paying attention to overall health status, as well as happiness and social support, should also be emphasized.

Additional education on complexity and uncertainty, as well as the limitations of molecular understandings of cancer, should also be extended to researchers and physicians. Some leaders in the field have already admitted this issue: 

“We lack the conceptual paradigms and computational strategies for dealing with this complexity. Additionally, equally painful, we do not know how to integrate individual datasets, such as those deriving from cancer genome analyses, with other, equally important datasets, such as proteomics. This is most frustrating, since it is becoming increasingly apparent that a precise and truly useful understanding of the behavior of individual cancer cells and the tumors that they form will only come once we are able to integrate and then distill these data.” [2].

Clearly, any new conceptual paradigms must include genome- and information-based evolutionary theories. It is thus timely to introduce different theories of cancer to physicians, who can provide feedback based on clinical realities. It is crucial to understand that the same treatment can be good or bad for different individuals. Finally, the issue of overdiagnosis and overtreatment will also be considered, and the question of living with cancer rather than trying everything to kill all cancer cells should be addressed.

As we are writing, there are many news reports, official commemorations, and editorial comments from research journals celebrating the 50-year milestone of the war on cancer. Most are excited about the potential “golden age” of cancer treatment given our enormous body of molecular and genetic knowledge, where every tumor has a unique signature which is personalized targets [133,134]. We share the excitement, but caution that only the correct ideas and strategies will deliver.

Fifty years is a long time. We hope that the next 50 years will witness changes in attitudes towards cancer, with solid theories that will improve ideas and outcomes [3,8].

## Figures and Tables

**Table 1:** Examples of alternative theories/concepts for explaining cancer.

Examples of Alternative Theories/Concepts for Explaining Cancer		Ref.
**Aneuploidy Theory**	Duesberg et al. (1998)	[25]
	Duesberg and Rasnick (2000)	[26]
	Gibbs (2003)	[27]
	Weaver and Cleveland (2007)	[28]
	Pavelka et al. (2010)	[29]
	Siegel and Amon (2012)	[30]
	Ye et al. (2018)	[31]
**Tissue organization field theory (TOFT)**	Soto and Sonnenschein, (2011)	[32]
	Baker (2011)	[33]
	Soto and Sonnenschein (2013)	[34]
**Cancer attractor theory**	Huang et al. (2009)	[35]
	Huang (2013)	[36]
	Kulkarni et al. (2013)	[37]
**Endogenous network hypothesis**	Ao et al. (2010)	[38]
**Physiological regulatory networks**	Noble (2021)	[39]
**Cancer represents a type of atavism**	Davies (2021)	[40]
**Retrotransposon-mediated genome evolution**	Wilkins (2010)	[41]
**Human genome mismatches the changing environment**	Gluckman (2011)	[42]
**Cancer represents a trade-off of adaptation and survival**	Horne et al. (2014)	[43]
	Heng (2015), (2019)	[3,8]
**Epigenetic alterations drive cancer**	Feinberg et al. (2006)	[44]
**Physical or chemical triggers**	Jaffe (2005)	[45]
	Levin (2021)	[46]
**Infection**	Ewald (1998)	[47]
**The Warburg effect and** **metabolic contribution**	Warburg (1956)	[48]
**Mutator phenotype**	Loeb (1974)	[49]
**Heterogeneity and tumor society, fuzzy inheritance**	Heppner (1984), Heppner and Miller (1988)	[50,51]
	Heng (2015)	[3]
**The first cell**	Raza (2019)	[52]
**New system emergent from** **system constraints**	Heng and Heng (2021)	[53]
**Complex adaptive system theory, chaotic systems**	Heng (2006), (2013), (2019) Tez 2016	[8,54,55] [56] [57]
**Extrachromosomal DNA**	Wu et al. (2019)	[58]
**Illegitimate genomic integration of cell-free chromatin**	Raghuram et al., (2019)	[59]
**Cancer as a new cellular species**	Huxley (1956)	[60]
	Van Valen (1991)	[61]
	Duesberg and Rasnick (2000)	[26]
	Ye et al. (2007)	[62]
	Heng (2007)	[63]
	Vincent (2010)	[64]
	Heng (2015)	[3]
	Bloomfield and Duesberg (2016)	[65]
	Paul (2021)	[66]
**Chaotic genomes (structural and numerical subtypes):** Massive karyotype reorganization; Polyploidy giant cancer cells; Micronuclei clusters; Chromosome fragmentations; Anstasis; and other overlooked chromosomal and nuclear variations	Walen (2010)	[67]
	Erenpreisa et al. (2005) (2020)	[68,69]
	Heng et al. (1988), (2006), (2008), (2013)	[6,54,55,70]
	Zhang et al. (2014)	[71]
	Niu et al. (2016)	[72]
	Chen et al. (2019)	[73]
	Liu (2018)	[74]
	Liu (2020)	[75]
	Ye et al. (2019)	[76]
	Zaitceva et al. (2021)	[77]
	Stevens et al. (2007)	[78]
	Pienta et al. (2020)	[79]
**Punctuated macroevolution**	Heng et al. (2006)	[6]
	Navin et al. (2011)	[80]
	Sottoriva et al. (2015)	[81]
	Shapiro (2021)	[82]
	Shapiro and Noble (2021)	[83]
	Furst (2021)	[84]
	Vendramin et al. (2021)	[85]

## Data Availability

Not applicable.
